# Sulfur redox mediator for low-temperature flexible amorphous oxide CMOS electronics

**DOI:** 10.1126/sciadv.adz6914

**Published:** 2025-10-24

**Authors:** Mingyang Wang, Taoyu Zou, Youjin Reo, Yong-Sung Kim, Min Gyu Kim, Minwoo Choi, Yongyoung Park, Kiyeon Yang, Youngjae Kang, Huihui Zhu, Yong-Young Noh, Ao Liu

**Affiliations:** ^1^ Institute of Fundamental and Frontier Sciences, State Key Laboratory of Electronic Thin Films and Integrated Devices, University of Electronic Science and Technology of China, Chengdu 611731, China.; ^2^ Department of Chemical Engineering, Pohang University of Science and Technology; Pohang, Gyeongbuk 37673, Republic of Korea.; ^3^ Korea Research Institute of Standards and Science, Daejeon 34113, Republic of Korea.; ^4^ Beamline Research Division, Pohang Accelerator Laboratory, Pohang University of Science and Technology, Pohang 37673, Republic of Korea.; ^5^ Thin Film Technical Unit, Samsung Advanced Institute of Technology, Samsung Electronics, Suwon-Si 16678, South Korea.; ^6^ School of Physics, University of Electronic Science and Technology of China, Chengdu 611731, China.

## Abstract

Amorphous p-type oxides are essential for next-generation flexible and scalable complementary metal-oxide semiconductor (CMOS) technologies. Among emerging candidates, tellurium-based oxides (Te-TeO*_x_*) show great promise due to their unique Te-Te conduction networks embedded within the amorphous TeO_2_ matrix. However, controlled formation of these conduction channels remains challenging, limiting both hole transport and dopability under low thermal budgets. Here, we report a sulfur-mediated redox strategy that modulates the local bonding environment via TeO_2_ dissociation and partial Te^4+^ reduction, promoting formation of short-chain Te-Te networks. This enables high-performance p-channel thin-film transistors processed at an ultralow temperature of 120°C, exhibiting an average hole mobility of 11.5 cm^2^ V^−1^ s^−1^ and on/off current ratios around 10^6^ with high uniformity and reproducibility. Integration with n-type counterparts enables all-oxide CMOS circuits on both flexible and rigid substrates, including inverters with gain up to 1694, ring oscillators operating at 339 kHz, and large-scale functional circuits with rail-to-rail output.

## Teaser

Sulfur redox strategy enables low-temperature fabrication of p-type oxide thin-film transistors and flexible integrated circuits.

## INTRODUCTION

Amorphous n-type oxide semiconductors offer high electron mobility, large-area uniformity, and precise tunability of electrical conductivity and carrier concentrations at low temperatures, advantages absent in other amorphous semiconductors such as silicon (*1*, *2*). Progress in this class of materials has driven substantial advances in thin-film transistor (TFT), enabling applications ranging from flat-panel displays to the Internet of Things, in-memory computing, microprocessors (*3*–*9*). In contrast, the lack of high-performance p-type oxides remains a major bottleneck for the development of complementary metal-oxide semiconductor (CMOS) technologies, which are foundation of modern analog and digital integrated circuits (ICs) (*10*, *11*). Amorphous or poorly crystallized p-type oxides often suffer from adequate semiconducting properties and limited hole doping efficiency (*12*).

Research on p-type oxide semiconductors have long been limited by low hole concentrations, poor mobilities, and the need for rigorous and high-synthesis temperatures (>300°C). Polycrystalline tin monoxide (SnO) is a leading candidate for low-temperature, high-mobility p-channel TFTs and CMOS ICs (*13*–*16*). The efforts achieve TFT hole mobilities more than 10 cm^2^ V^−1^ s^−1^ with inverter gains up to 765 and oscillator frequencies of 77 kHz (*14*, *17*, *18*). The key challenge for TFT application is the relatively low on/off current ratio (*I*_on_/*I*_off_ ≈ 10^4^), arising from the electron transport via Sn^4+^ states. The polycrystalline microstructures also limit uniformity, scalability, and device downscaling in mass production (*4*). Alternatives such as elemental tellurium (Te) (*19*, *20*), carbon nanotubes (*21*), transition-metal dichalcogenides (*22*, *23*) and halide perovskites (*24*–*26*) offer unique advantages but face challenges in stability, uniformity, process complexity, and industrial compatibility. Recently, Te-based oxides (Te-TeO*_x_*) have emerged as a promising solution (*27*–*32*), which uses spontaneous TeO_2_ decomposition to form high-mobility Te-Te channels embedded within an amorphous TeO_x_ matrix. Their scalability, stability, and uniformity have realized monolithic three-dimensional (3D) CMOS integration, showing strong potential for practical applications (*33*, *34*).

The development of functional channel layers with controllable semiconducting properties has been a long-standing research focus in oxide electronics. Previous work showed that Se alloying in Te-TeO*_x_* system can realize lower hole carrier concentration and enhanced TFT performance (*27*) but still requires elevated postannealing temperatures to achieve sufficient Te-Te/Te-Se network connectivity. To date, an effective strategy for simultaneously enriching hole-conduction channels and achieving doping in amorphous p-type oxides at low temperatures remains lacking. Here, we present a sulfur (S)–mediated redox strategy that promotes in situ formation of Te conduction channels and hole doping in Te-TeO*_x_* systems, achieving high-performance, stable p-channel TFTs at low processing temperatures (~120°C). Integration with n-type InGaZnO TFTs enables diverse all-oxide CMOS ICs on both rigid and flexible substrates with high functionality and yield.

## RESULTS

### The mechanism of sulfur mediator

**Fig. 1.**
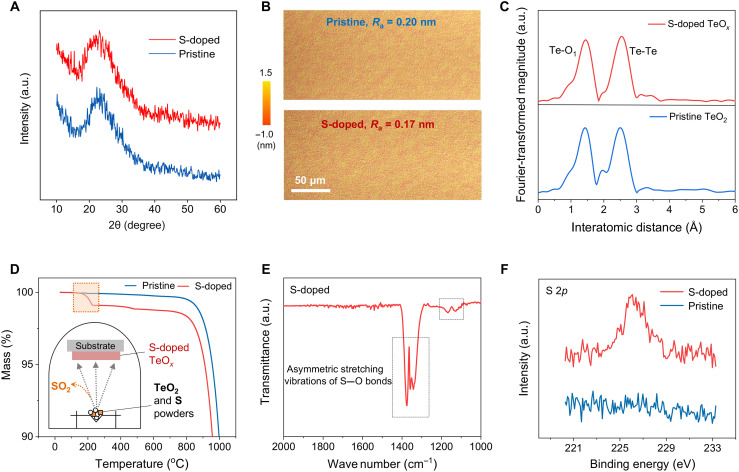
Film characterizations and redox mechanism analysis of amorphous S-doped TeO*_x_*. (**A**) XRD spectra of the pristine and S-doped TeO_x_ thin films. (**B**) Surface topography of the corresponding TeO*_x_*-based thin films. (**C**), Fourier transforms of the Te K-edge *k*3-weighted EXAFS spectra of the pristine and S-doped TeO*_x_* samples. (**D**) TGA curves of the pristine TeO_2_ powder and TeO_2_:S mixture. (**E**) FTIR spectrum of the vapor products of the TeO_2_:S mixture at 240°C. (**F**) XPS spectra of the S 2*p* states of the pristine and S-doped TeO*_x_* films. a.u., arbitrary unit.

The TeO*_x_*-based thin films were deposited via thermal evaporation, followed by low-temperature (120°C) annealing in air. Sulfur incorporation was achieved by mixing TeO_2_ and S powder in a precise weight ratio, followed by evaporation in a tungsten boat (see Materials and Methods). To investigate the effects of S doping, we tested various S blending ratios and determined the optimal S/Te weight ratio of S-doped films in this work to be 4.8% using high-resolution inductively coupled plasma mass spectrometry. We first conducted a structural analysis using x-ray diffraction (XRD), with the results showing the amorphous-like nature of the deposited TeO*_x_*-based films, indicating that S doping has a negligible disruption on the TeO*_x_* structure (Fig. 1A). The amorphous structure of the films provides a smooth and uniform surface morphology, with average roughness values of 0.20 (±0.02) nm (Fig. 1B). The film densities were measured to be ~5.0 g cm^−3^, with a thickness of ~15 nm, as determined by x-ray reflectivity analysis (fig. S1).

To investigate the detailed component information of the films, we measured their Te K-edge extended x-ray absorption fine structure (EXAFS) spectra. The Fourier transforms of the EXAFS spectra for both pristine TeO*_x_* and S-doped samples (TeO_x_:S) exhibit only short Te─O_1_ bonds, with no evidence of longer Te─O_2_ and long-range ordered Te─O─Te bonding, indicating their amorphous nature (Fig. 1C and fig. S2). The x-ray absorption near-edge structure (XANES) spectra show that the samples are primarily composed of a mixture of Te and TeO_2_. The S-doped TeO*_x_* sample exhibits lower Te─O coordination and higher Te─Te coordination, with a Te/TeO_2_ composition ratio of 0.28/0.72, than the pristine TeO*_x_* sample (Te/TeO_2_ = 0.21/0.79) (fig. S3 and table S1). Because Te─Te connections serve as the primary pathways for hole percolation in TeO*_x_*, S incorporation is expected to improve the overall electrical properties of the TeO*_x_*-based films and devices.

Notably, the as-evaporated TeO*_x_*:S also shows an increase in Te/TeO_2_ ratio, indicating that S-facilitated Te generation occurs during the evaporation process. Owing to the strong bonding between S and O, it is speculated that elemental S acts as a reduction mediator, dissociating TeO_2_ and forming elemental Te. Thus, elemental S is oxidized and combined with dissociated O^2−^ to generate SO_2_ through the reaction: S + 2O^2−^ + Te^4+^ → Te + SO_2_↑. To further verify this hypothesis and analyze the evaporation process, we performed thermogravimetric analysis coupled with Fourier-transform infrared spectroscopy (TGA-FTIR) on a TeO_2_/S powder mixture in an inert environment and collected the generated vapor for analysis. Compared with the pristine TeO_2_ sample, the S-mixed sample exhibits a notable weight loss at ~250°C (Fig. 1D). The infrared analysis of the reaction gas collected at this temperature revealed the characteristic absorption peaks of SO_2_ (Fig. 1E). We also confirmed that this weight loss is not attributable to the evaporation of elemental S, given its boiling point of ~450°C. This finding is supported by the corresponding weight loss observed when additional elemental S is added (fig. S4).

**Fig. 2.**
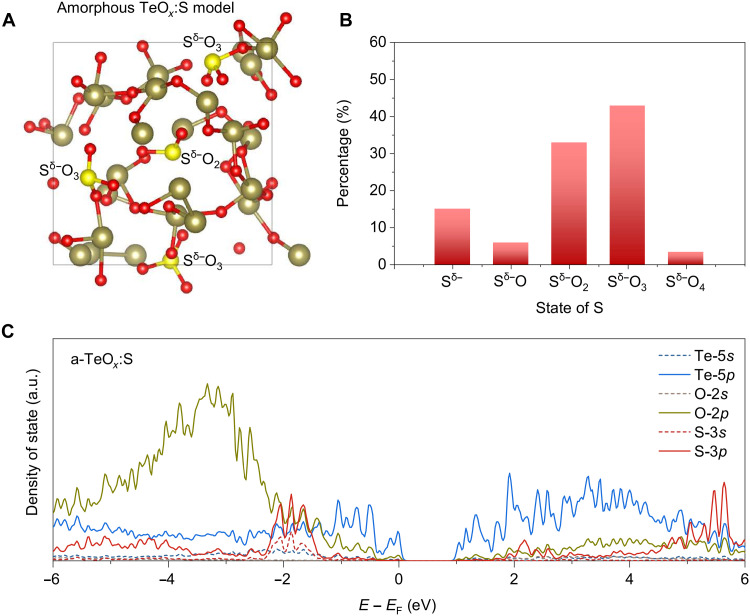
Atomic and electronic structures of amorphous S-doped TeO*_x_*. (**A**) Atomic structure of amorphous S-TeO*_x_* generated via DFT calculations. (**B**) Distribution of anionic S states as identified in the DFT calculations. (**C**) Projected density of states of the S-3*s*, S-3*p* (red), Te-5*s*, Te-5*p* (blue), and O-2*s* and O-2*p* (yellow and brown) states in S-TeO*_x_*. The annotated peak in the conduction band is associated with the partially empty S_3*p*_^δ−^ (0 ≤ δ < 2) states of S^δ−^O*_x_*.

To determine whether S is partially incorporated into TeO*_x_*, secondary ion mass spectrometry and x-ray photoelectron spectroscopy (XPS) were performed. The 3D elemental mapping image shows that S atoms are uniformly distributed through the film (fig. S5). The XPS analysis of the S 2*p* spectra in Fig. 1F indicates that S acts as an anion in TeO*_x_* (*35*). To better understand the role of S and its chemical state, density functional theory (DFT) calculation was performed. The DFT results reveal five plausible anionic S^δ−^ states in TeO*_x_*: fourfold S^δ−^O_4_, threefold S^δ−^O_3_, twofold S^δ−^O_2_, onefold S^δ−^O, and zerofold S^δ−^ configurations, where δ is in the range of 0 ≤ δ < 2. Because the S^δ−^─O^2−^ covalent bond is stronger than the Te^4+^─S^δ−^ ionic bond, S is dominantly found in S─O bonds or an isolated S state, rather than in Te─S bonds (Fig. 2A). The zerofold S^δ−^, which can be configured as a Te─S bond, is less than 15%. This finding is also consistent with the XANES analysis, which shows no obviously detectable Te─S bonds. DFT calculations on 60 different models show that the most prevalent configuration in the molecular structure is threefold S^δ−^O_3_ (42.9%) (Fig. 2B). This configuration consists of partially empty S 3*p* states in the conduction band, with anionic S^δ−^ forming covalent bonds with O^2−^ (Fig. 2C). As the reaction proceeds, a portion of the anionic S^δ−^ donates electrons to cationic Te^4+^, leading to the formation of cationic S^4+^, which then produces SO_2_ gas and generates additional metallic Te^0^, a species that is crucial for improving hole transport in amorphous TeO*_x_*.

The redox mediator mechanism of S revealed in this study exhibits a fundamental difference from its conventional role in passivating oxide vacancies in n-type oxide semiconductors (*36*). The mechanism also differs from the selenium alloying strategy (*27*), where performance enhancement relies on suppressing bulk conductivity in the Te conduction channel while requiring relatively high-temperature annealing to establish Te-Te/Te-Se connections for charge transport. In contrast, our S-mediated redox approach enables in situ formation of short-chain Te-Te during evaporation, directly embedding them into the TeO_2_ matrix without requiring stringent postannealing. Notably, simply codepositing Te and TeO_2_ fails to achieve desired performance, as uncontrolled formation of long-chain Te results in high off current. Our findings establish a versatile strategy for modulating hole transport channels and doping via strong redox mediators (extendable beyond S), offering a previously unknown design principle for engineering TeO*_x_*-based semiconductors across diverse applications.

### TFT and CMOS IC characterizations

**Fig. 3.**
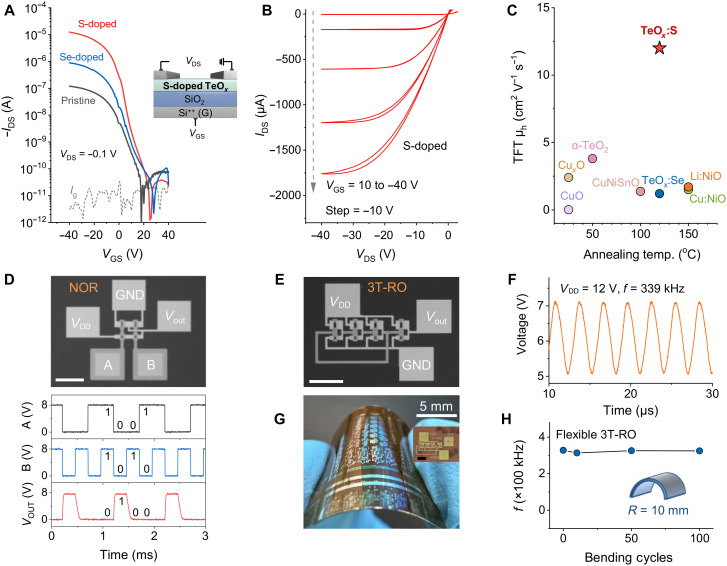
Electrical characterizations of p-channel S-doped TeO_x_ TFTs and small-scale all-oxide–based CMOS ICs. (**A**) Transfer curves of the pristine, TeO*_x_*:Se, and TeO*_x_*:S TFTs annealed at 120°C (*V*_DS_ = −0.1 V; inset: device structure). (**B**) Output curve of the TeO*_x_*:S TFT annealed at 120°C. (**C**) Benchmark of the mobility of representative oxide TFTs fabricated at low temperatures. (**D**) Image and output waveform of the CMOS NOR gate based on the p-type TeO*_x_*:S and n-type IGZO TFTs. (**E**) Image and (**F**) output waveform of the three-stage RO on Si/SiO_2_ substrate. (**G**) Image and (**H**) oscillation frequency at different bending times of the flexible three-stage RO on PI. All scale bars in the microscopy images denote 150 μm. All the IGZO and TeO*_x_*:S devices using in CMOS ICs are fabricated on 20-nm Al_2_O_3_ dielectrics.

To verify the feasibility of TeO*_x_*:S as channel layers for low-temperature, high-performance TFTs, the bottom-gate, top-contact device was fabricated on SiO_2_ dielectric, with Ni as source/drain electrodes and a post annealing temperature of 120°C. The transfer characteristics of pristine TeO*_x_*, Se-alloyed TeO*_x_* (TeO_x_: Se), and TeO*_x_*:S (4.8%) TFTs are shown in Fig. 3A (fig. S6 shows the dual-sweep transfer curves of the TeO*_x_*:S TFT). The hump-like transfer curves may result from the formation of multicomponent in the Te-TeO*_x_* channel (i.e., Te and TeO*_x_*), leading to multiple conduction pathways. The pristine TeO*_x_* TFT exhibit poor p-channel behavior, with a low field-effect hole mobility (μ_h_) of 0.1 cm^2^ V^−1^ s^−1^ and an on/off current ratio (*I*_on_/*I*_off_) of ~10^4^. The Se alloying enhances μ_h_, reaching ~1.2 cm^2^ V^−1^ s^−1^. In contrast, the TeO*_x_*:S TFT show notably improved characteristics, achieving a high μ_h_ of 12 cm^2^ V^−1^ s^−1^, a high *I*_on_/*I*_off_ of ~10^6^, a subthreshold swing (*SS*) value 3.5 V dec^−1^, a threshold voltage (*V*_TH_) of −2.5 V, and good uniformity over a 4-inch wafer with an average μ_h_ of 11.5 cm^2^ V^−1^ s^−1^ (fig. S7). The optimal TeO*_x_*:S TFT demonstrates satisfactory operational stability during long-term continuous on/off switching and bias-stress tests (fig. S8) and robust air stability with negligible performance degradation after 3 months of storage in ambient air without encapsulation (fig. S9). The output curves show good current linearity at low source-drain voltages and saturation at high source-drain voltages, indicating ohmic contact between the channel layer and electrodes (Fig. 3B). The electrical characterizations of 120°C annealed channel layers using Hall measurement show the same trend: Pristine TeO*_x_* has low mobilities of ~3 cm^2^ V^−1^ s^−1^ with hole concentrations of ~10^16^ cm^−3^, while the incorporation of S greatly increases the Hall mobility to around 20 cm^2^ V^−1^ s^−1^, with the enlarged hole concentration of ~10^17^ cm^−3^ (fig. S10).

Typically, p-type oxides exhibit inherently poor hole transport. The deposited channels require relatively high processing temperatures to achieve dense or well-crystallized structures for adequate device performance. In this study, S-mediated Te phase formation in the TeO*_x_* composite channel creates sufficient holes and conduction paths, enabling superior electrical performance even at low post-treatment temperatures (Fig. 3C). The flexible modulation of the high-mobility Te composition in this hybrid oxide system endows TeO*_x_* with the ability to meet different application requirements and offers unique insights into the design of dopable and high-mobility amorphous p-type oxide-based semiconductors.

To further demonstrate the viability of establishing fully oxide-based CMOS technologies, we constructed functional ICs comprising well-matched n-channel InGaZnO (IGZO) and p-channel TeO*_x_*:S TFTs on 20-nm Al_2_O_3_ gate dielectrics (fig. S11). The n-IGZO TFT shows a field-effect electron mobility of 13 cm^2^ V^−1^ s^−1^, an *I*_on_/*I*_off_ of more than 10^5^, and a *V*_TH_ of 1.1 V. The p-TeO*_x_*:S counterpart shows a μ_h_ of 11 cm^2^ V^−1^ s^−1^, an *I*_on_/*I*_off_ of over 10^5^, and a *V*_TH_ of −0.9 V. Benefitting from the well-matched complementary TFTs, the CMOS inverter exhibits an ultrahigh gain of 1694 at a *V*_DD_ voltage of 8 V, which is superior to reported all-oxide CMOS inverters (table S2). The inverter also shows a high noise margin of 3.36 V, achieving the 84% of the ideal value of *V*_DD_/2, indicating its strong noise immunity (fig. S12). In addition, we integrated the logic gates including inverters, not-or (NOR), not-and (NAND), and exclusive-or (XOR), ring oscillators (ROs), arithmetic units (full adders), and sequential circuits (flip-flops and counters). These are the fundamental components of a central processing unit and are responsible for arithmetic and logical operations and data storage and clock generation.

The examination of the dynamic performance of logic gates demonstrates correct Boolean logic functions and rail-to-rail characteristics at an input signal frequency of 2 kHz (Fig. 3D). To evaluate the speed of the CMOS circuits, we fabricated ROs by cascading inverters and incorporating an additional inverter as an output buffer to minimize interference (Fig. 3E). At *V*_DD_ = 12 V, the RO achieves a high oscillation frequency (*f*) of 339 kHz and a fast stage delay (τ) of 0.49 μs, corresponding to a stage-switching frequency of up to 2.04 MHz (Fig. 3F). The high *f* and fast τ values of our devices surpass those of previously reported oxide-based ROs (table S2). The low-temperature processing also benefits the integration of flexible electronics, which are essential for the Internet of Things and lightweight wearable applications (*37*). To evaluate device performance on a flexible substrate, we fabricated the RO on a polyimide (PI) substrate (Fig. 3G), where it maintained a high *f* of 328 kHz and fast τ of 0.51 μs (fig. S13). Owing to the homogeneity and absence of grain boundaries in the amorphous films, our flexible RO sustained a high *f* of 325 kHz with less than 1% degradation after hundreds of bending cycles at a 10-mm radius of curvature (Fig. 3H). This overall performance surpasses that of previously reported flexible all-oxide CMOS and pseudo-CMOS ROs (*f* ≈ 20 kHz at *V*_DD_ = 10 V) (*38*, *39*).

**Fig. 4.**
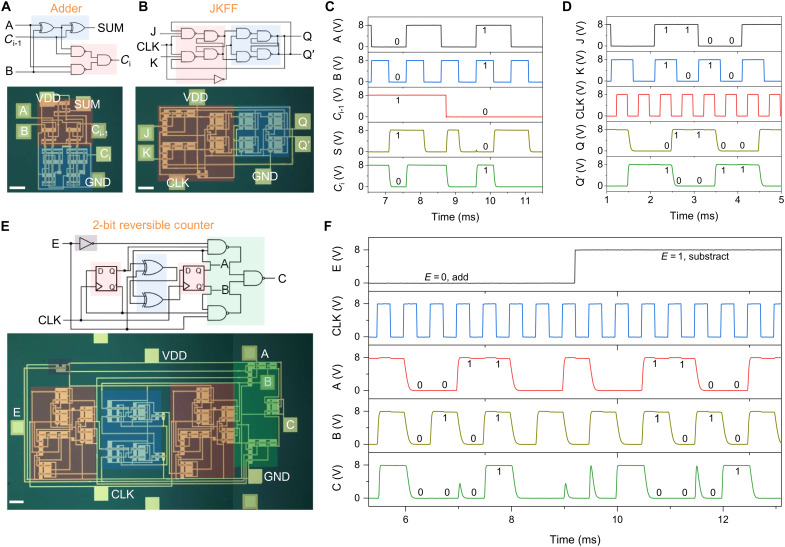
Electrical characterizations of functional large-scale all-oxide–based CMOS ICs. Circuit schematics and optical images of the (**A**) 1-bit full adder and (**B**) JKFF. Dynamic input-output waveforms of the (**C**) 1-bit full adder, and (**D**) JKFF. (**E**) Circuit schematic and optical image and (**F**) dynamic input-output waveforms of the 2-bit reversible counter. All scale bars denote 150 μm.

The demonstration of high-performance small-scale ICs highlights the processability and compatibility of low temperature–processed TeO*_x_*:S in CMOS ICs construction. The unique uniformity of the amorphous semiconductors also supports the integration of large-scale ICs. To demonstrate this application, we fabricated a 1-bit CMOS full adder consisting of 32 TFTs and a Jack Kilby flip-flop (JKFF) consisting of 38 TFTs, respectively (Fig. 4, A and B). Figure 4C shows the dynamic input-output characteristics of the full adder, where input voltages of 0 and 8 V correspond to logic “0” and “1”, respectively. For all possible input combinations, including a consideration of the stored data of the previous bit (*C*_i-1_), our adder achieves correct sum (*S*) and carry (*C*_i_) outputs with rail-to-rail operation, thus meeting the requirements for arithmetic circuits. The JKFF is the most versatile flip-flop circuit that is capable of forming all other types of flip-flop circuits for data storage and synchronization within sequential logic systems. Our JKFF could be triggered by falling edge of clock (CLK) signals, exhibiting correct logic functions, including flip-flop, set to 0, set to 1, and hold, with ideal rail-to-rail outputs for different input combinations, and achieving reliable 1-bit data storage and transmission (Fig. 4D).

To achieve the higher-scale IC, we further fabricated a 2-bit reversible counter consisting of 90 TFTs, including two delay flip-flops (DFFs), two XORs, and several logic gates (Fig. 4, E and F). Reversible counters are widely used in computer systems for address generation, time counting, and cycle control, rendering them one of the most important sequential circuits in digital chips. The counter can dynamically switch between addition and subtraction modes based on the enable signal (E). When E is set to 0, the counter increments with each CLK signal, cycling A and B outputs from “00” to “11.” Upon reaching 11, the C port generates a carry signal, and A and B reset to 00 on the next CLK. When E is set to 1, the counter decrements, with the A and B outputs decreasing from 11 to 00, and the C port outputs a borrow signal at 00. The counter successfully realizes the reversible addition and subtraction counting function of 2-bit binary data through data transmission via flip-flops and logical operations using XOR gates. It also achieves perfect rail-to-rail output without logic errors with a 2-kHz CLK input signal input. To further improve the operational frequency of the integrated ICs, efforts in scale-down of device size, well-designed circuit physical layout and interconnection optimization can be effective. Furthermore, owing to the high uniformity and yield of the amorphous materials, all six reversible counters fabricated on a single chip worked, indicating a yield of at least 99.85% for the integrated devices.

## DISCUSSION

We have developed a redox-mediated strategy that used S to facilitate the deoxygenating of TeO_2_, modulating the formation of Te-Te hole-conduction channels and p-doping in amorphous TeO_2_, enabling the low-temperature, high-performance, and stable p-channel TFTs. By exploiting the high stability, robust processing compatibility, and large-area uniformity of the material, we demonstrated multifunctional all-oxide CMOS ICs on both rigid and flexible substrates. These include inverters, logic gates, ROs, full adders, flip-flops, and 2-bit reversible counters, all of which exhibited good performance and high yields. The inverter shows a record gain value of 1694, and the ROs exhibit a high oscillating frequency of 339 kHz. Moreover, logic gates and large-scale ICs demonstrate correct logical functionality with rail-to-rail output. The advancement in amorphous oxide-based CMOS technology lays the groundwork for the development of cost-effective, large-area, and flexible complementary electronics and microprocessors beyond traditional semiconductor technology.

## MATERIALS AND METHODS

### Thin-film fabrication and material characterizations

TeO_2_ (≥97%) and S (99.99%) powders were purchased from Sigma-Aldrich and directly used as evaporation sources. The TeO*_x_*:S films were deposited using a thermal evaporator by loading the mixed TeO_2_ and S powders (400 mg of TeO_2_ and 5 mg of S for the optimal ratio) in a tungsten boat. The substrate temperature was maintained at room temperature, and the vacuum pressure before evaporation was ~6 × 10^−6^ torr. Before the film deposition, the substrate shutter was opened first and then slowly heated to maintain the evaporation rate of 0.5 Å/s until reaching the needed thickness (monitored by an INFIC SQC-300 system with a quartz crystal oscillator). The deposited samples were annealed at 120°C for 30 min in ambient air for device fabrication. The crystal structures of the films were analyzed using XRD with Cu Kα radiation (Bruker D8 ADVANCE). The XPS analysis was performed using a PHI 5000 VersaProbe instrument (Ulvac-PHI, Tokyo, Japan). The film surface morphology was measured by white light interference confocal three-dimensional profilometer (Sensofar 3D). The Hall measurements (JouleYacht HET3) of the films were performed using the van der Pauw method with a 0.6-T magnet in the air condition.

The S-doping percentages were characterized using high-resolution inductively coupled plasma mass spectrometry (Thermo Element XR) by dissolving the deposited films in a HNO_3_- and HCl-mixed acid solvent accompanied by microwave digestion. Te K-edge x-ray absorption near edge structure spectra of S-doped TeO*_x_* films were collected on the BL10C beam line at the Pohang light source (PLS-II) with top-up mode operation under a ring current of 250 mA at 3.0 GeV. The thermal analysis of TeO_2_/S powders was characterized by thermogravimetry analyzer (Mettler TG/DSC 3) and infrared Fourier-transform spectrometer (Thermo Fisher Scientific 50iS50). TGA was carried out in a flowing nitrogen atmosphere (the flow rate was 20 ml/min), and the vapor products were transported to the infrared spectrometer by a vacuum pump for analysis.

### DFT calculation

The DFT calculations were performed using the Vienna ab initio simulation package code (*40*). Projector augmented wave pseudopotentials and a kinetic energy cut-off of 500 eV were used (*41*, *42*). The PBE exchange-correlation functional was used (*43*). The amorphous structure was modeled as a cubic supercell using melt-and-quench molecular dynamics simulations. A random initial structure was melted at 3000 K for 3 ps and then quenched to 0 K at a rate of −1 K fs^−1^. The residual forces were relaxed to less than 0.01 eV/Å. A single *k*-point at (1/4, 1/4, 1/4) in the cubic Brillouin zone was used. The molecular dynamics time step was set to 1 fs. The supercell volume was fixed during the molecular dynamic simulations. We generated 60 different TeS_0.17_O_1.67_ amorphous structures (samples) in 68-atom (Te_24_S_4_O_40_) cubic supercells, respectively, using melt-and-quench molecular dynamics simulations beginning with 60 different random initial structures. The calculated properties were the mean values obtained for the 60 samples. The supercell volume was fixed during the molecular dynamic simulations with a lattice constant of 10.7 Å in the cubic supercell, which corresponds to 5.192 g/cm^3^ in density. Electronic structures were obtained using the Γ-centered 2 × 2 × 2 *k*-point mesh and the hybrid functional of Heyd-Scuseria-Ernzerhof, with a mixing parameter of 0.25 and a screening parameter of 0.2 1/Å (*44*).

### Fabrication and characterization of devices and circuits

A heavily doped Si wafer (resistivity: 1 to 100 ohm·cm) with a 100-nm thermally grown SiO_2_ was used as the gate electrode and the dielectric layer. The S-doped TeO*_x_* channels were deposited on SiO_2_ as channel layers using the aforementioned procedure. The shadow mask was covered on the substrate to obtain the patterned channel layers. Ni source/drain electrodes (40 nm) were deposited with a shadow mask using thermal evaporation to construct a bottom-gate top-contact TFT. The channel length and width (*L*/*W*) were 100/1000 μm. The TFTs electrical properties were characterized using a Keithley 4200 SCS at room temperature in a N_2_ glove box. The TFT bias-stress stability and the CMOS circuit measurements were carried out in air condition. The Te-based oxides show robust air stability, and the test environment has minor effect on the device electrical characterizations (fig. S14). The TFT mobility was extracted by standard square-law mode using the formula $\mu_{\text{FE}}=\frac{L}{WV_{D}C_{\text{ox}}}\frac{\partial \;I_{D}}{\partial \;V_{G}}$ at linear regime. For the CMOS circuits integration, fig. S15 shows the process flow diagram. First, 50-nm Mo was deposited using magnetron sputtering and patterned as bottom gate electrodes (the patterning process was carried out by ultraviolet (UV) lithography and wet etching processes). Then, 20-nm Al_2_O_3_ was deposited by atomic layer deposition as the gate dielectric. The Al_2_O_3_ layer was patterned by UV lithography and inductively coupled plasma etching process to form through-holes for final electrical interconnection. A 20-nm IGZO was deposited by magnetron sputtering with a power of 90 W at pure-Ar atmosphere and patterned as the n-type channel. The *L*/*W* of IGZO and TeO*_x_*:S TFTs were 10/20 μm. A 40-nm Ni was deposited by thermal evaporation and patterned as source/drain electrodes and the connecting lines. For the flexible CMOS circuits, the substrates are 125-μm-thick PI films. First, the PI substrate was cleaned by ethanol, acetone, and water in turn and then treated with oxygen plasma to eliminate organic residue and improve its surface hydrophilicity. Then, a 4-μm-thick PI film was deposited on the PI substrate to improve the surface topography by using spin coating. Subsequently, the substrate was annealed at 150°, 200°, and 250°C for 30 min to cure the spin-coated PI film and release the participating stress. Then, 100-nm SiO_2_ was deposited by plasma-enhanced chemical vapor deposition as a buffer layer. The input-output properties of the CMOS circuits were characterized by oscilloscopes (Tektronix 4 series BMSO), function generators (Tektronix AFG1000), and dc power suppliers.

## Supplementary Materials

### This PDF file includes:

Figs. S1 to S15 Tables S1 and S2 References
