# Supplementary material for: Sulfur redox mediator for low-temperature flexible amorphous oxide CMOS electronics
Source: Sci Adv. 2025 Oct 24;11(43):eadz6914. doi: 10.1126/sciadv.adz6914 (PMC12551691; doi:10.1126/sciadv.adz6914)
Supplement: Supplementary file 1 — Figs. S1 to S15 Tables S1 and S2 References [file sciadv.adz6914_sm.pdf]

Science Advances
AAAS

Supplementary Materials for

Sulfur redox mediator for low-temperature flexible amorphous oxide CMOS electronics

Mingyang Wang *et al.*

Corresponding author: Huihui Zhu, hhzhu@uestc.edu.cn; Yong-Young Noh, yynoh@postech.ac.kr; Ao Liu, ao.liu@uestc.edu.cn

Sci. Adv. **11**, eadz6914 (2025)
DOI: 10.1126/sciadv.adz6914

This PDF file includes:

Figs. S1 to S15
Tables S1 and S2
References

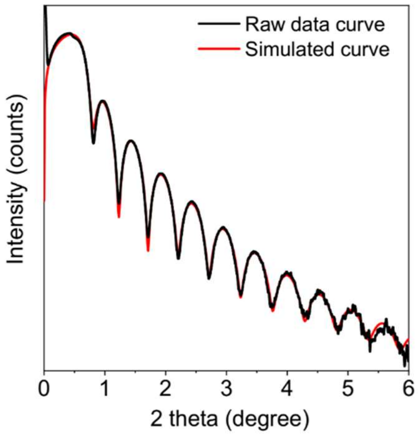

Fig. S1. X-ray reflectivity spectrum and fitting curve of the S-doped TeO_x thin film.

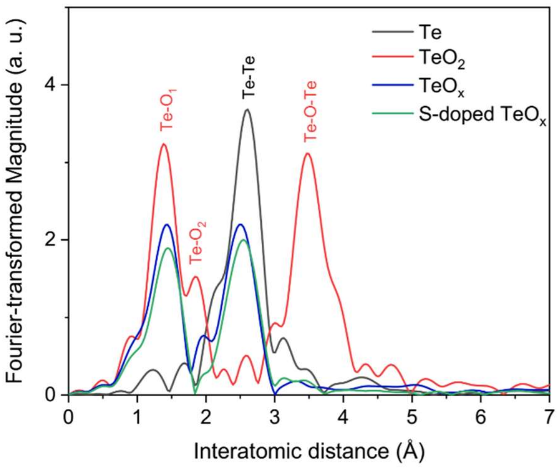

Fig. S2. Fourier-transformed magnitude of TeO_x, S-doped TeO_x, and reference Te, TeO_2.

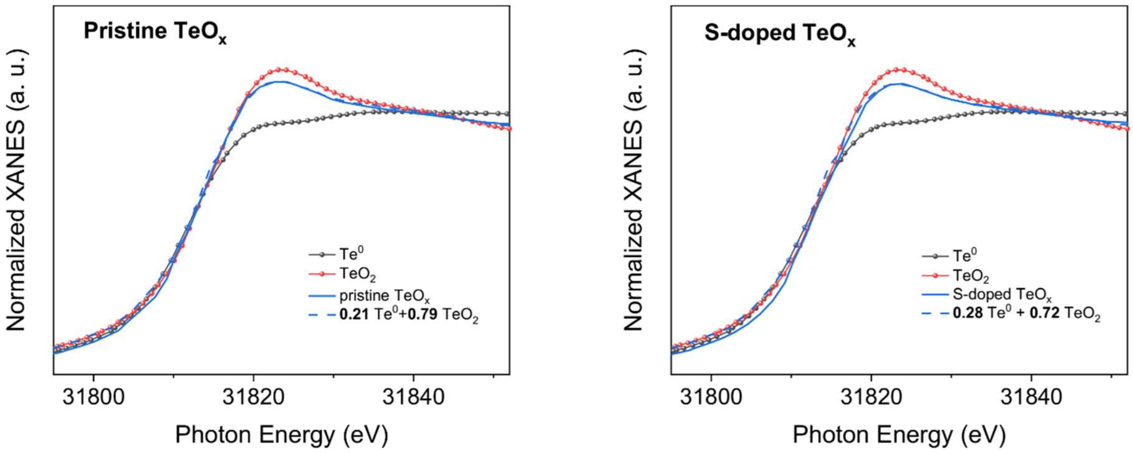

Fig. S3. XANES spectra of TeO_x and S-doped TeO_x samples and linear combination with references Te and TeO_2.

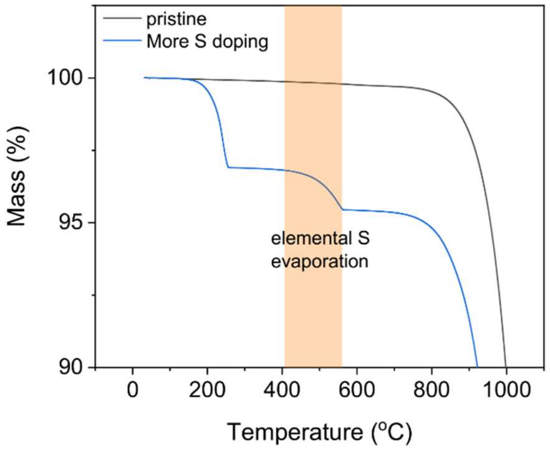

Fig. S4. Thermogravimetric curves of pristine TeO_2 powder and TeO_2/S (with higher S content) mixture.

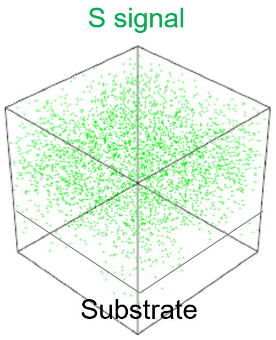

Fig. S5. 3D SIMS sulfur element mapping image of S-doped TeO_x thin film.

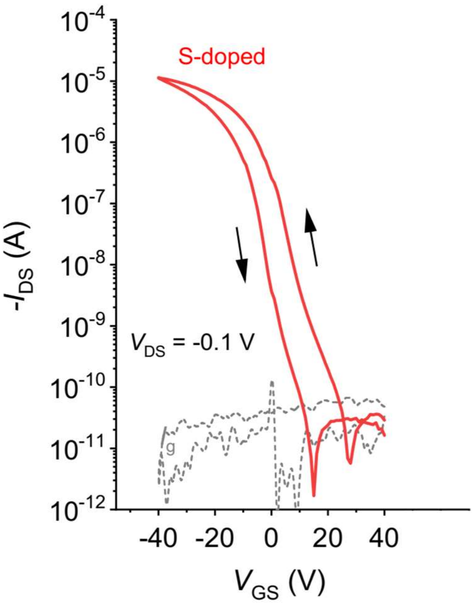

Fig. S6. Dual-sweep transfer curve of the TeO_x:S TFT.

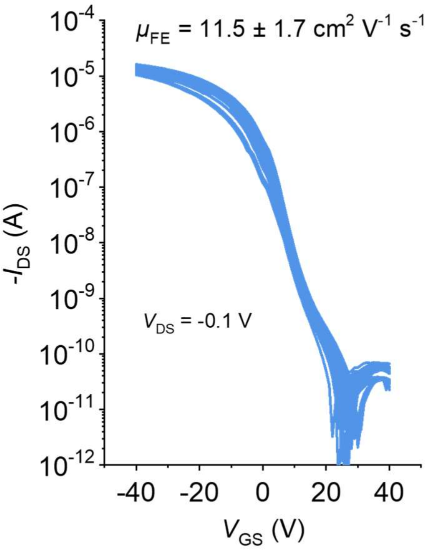

Fig. S7. Transfer curves of 25 devices on a 4-inch wafer.

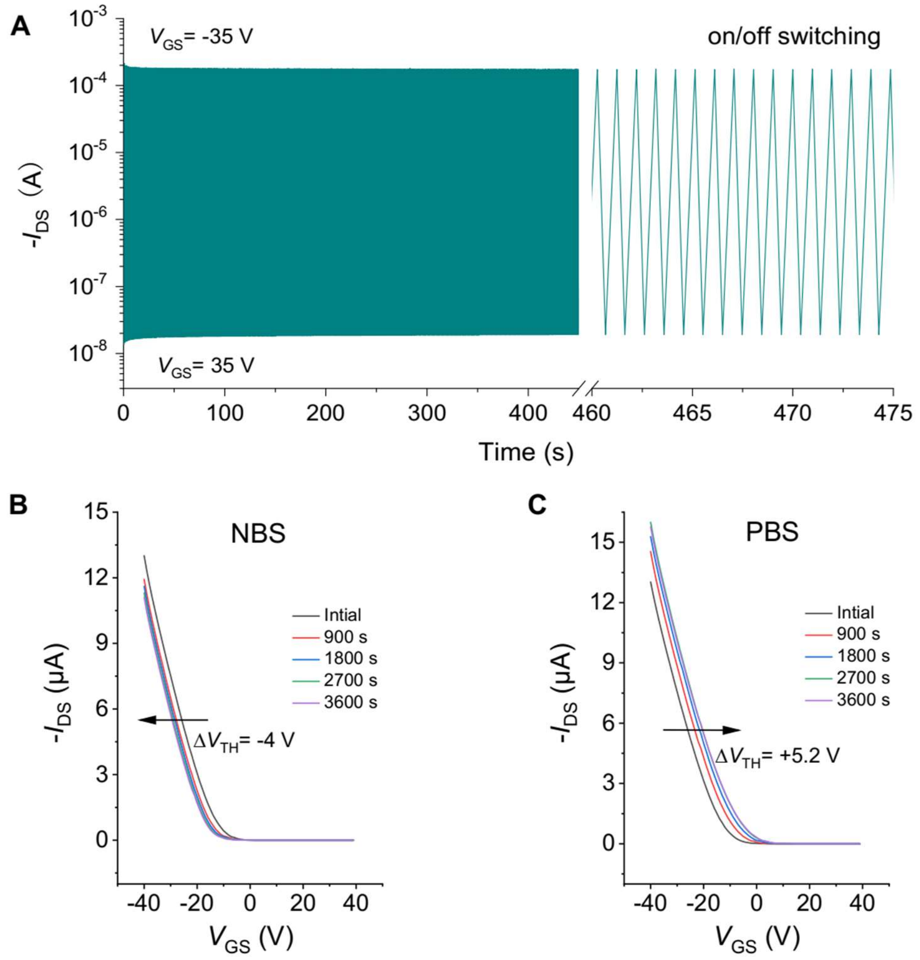

Fig. S8. Operation and bias-stress stability of one S-doped TeO_x TFT measured in air. (**A**), Continuous on/off switching test result (500 cycling). (**B**), Negative-bias stress and (**C**), positive bias stress test results ($V_{GS/DS}$ = -40 V).

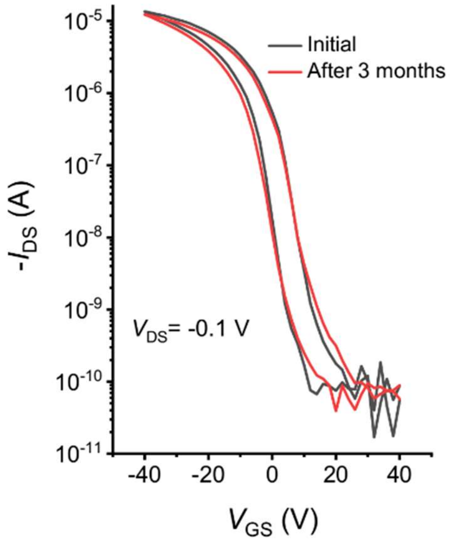

Fig. S9. Transfer curves of the fresh TeO_x:S TFT and stored in air for 3 months.

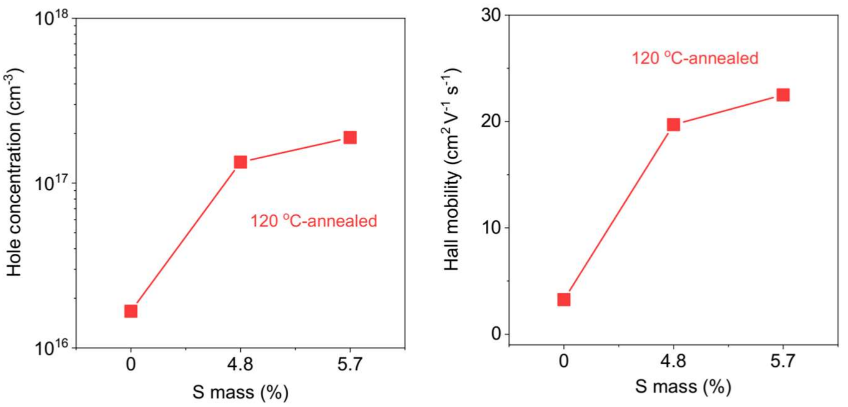

Fig. S10. Hall hole concentration and mobility as a function of S mass ratio.

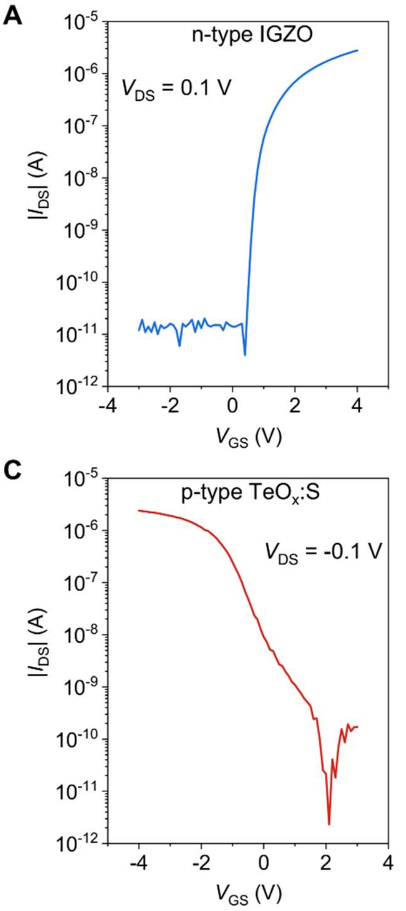

Fig. S11. Electrical properties of the complementary TFTs on 20 nm ALD Al_2O_3 dielectric using in CMOS ICs. (**A**), Transfer curve and (**B**), output curves of the n- IGZO TFT. (**C**), Transfer curve and (**D**), output curves of the p-TeO_x:S TFT.

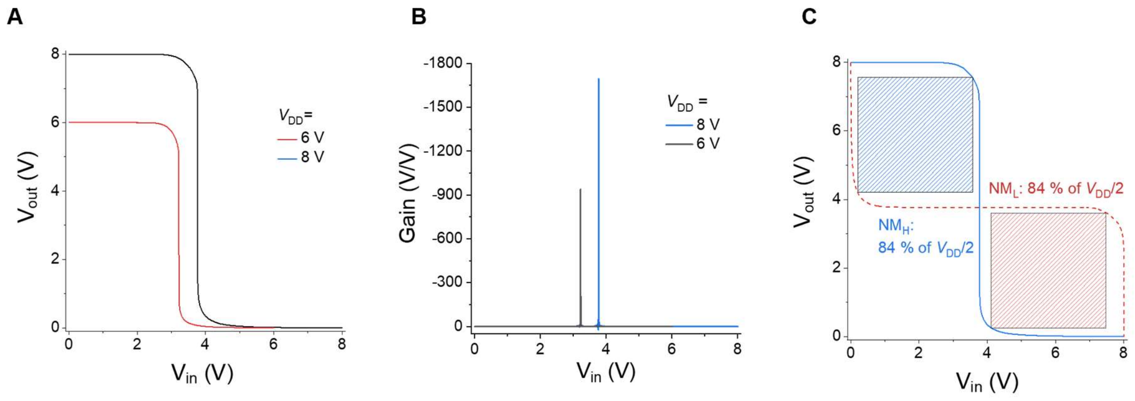

Fig. S12. Electrical properties of the CMOS inverter. (**A**), Voltage transfer characteristics and (**B**), the gain curve of the CMOS inverter. (**C**) Noise margin of the CMOS inverter at V_{DD} = 8 V.

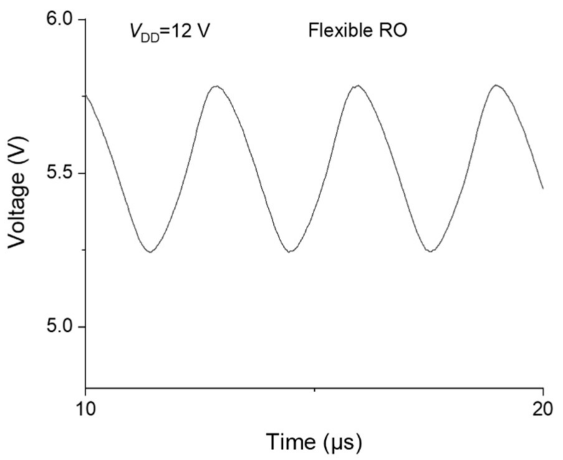

Fig. S13. Output waveform of the flexible 3-stage RO at V_{DD}=12 V.

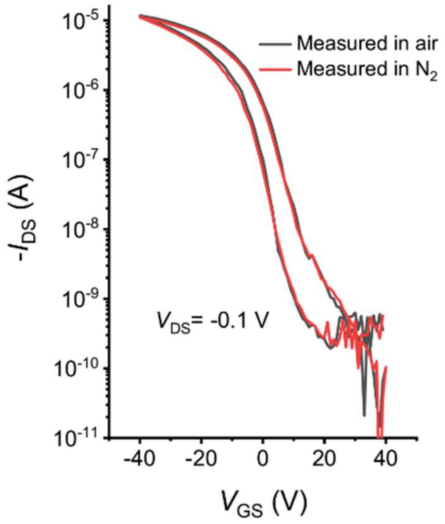

Fig. S14. Transfer curves of the TeO_x:S TFT measured in air and N_2.

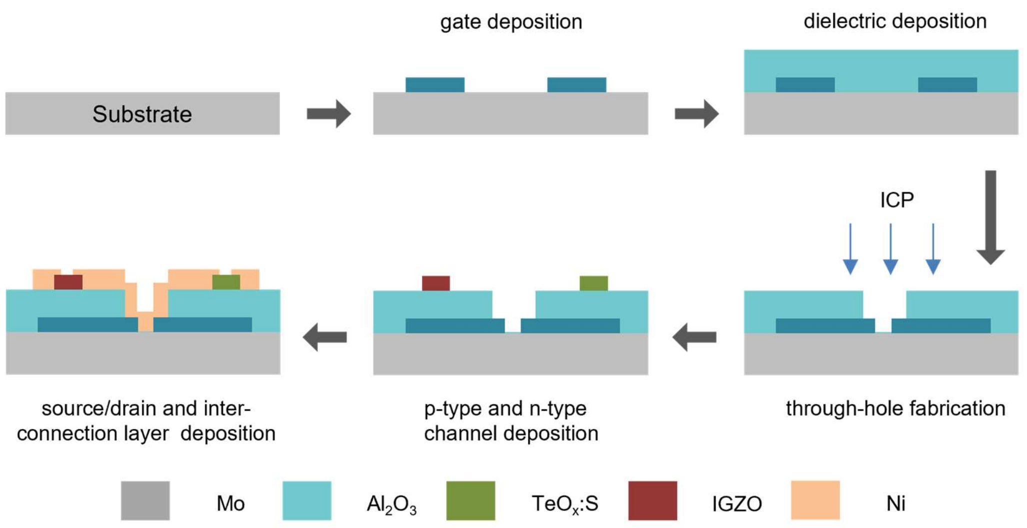

Fig. S15. Process flow diagram of the CMOS IC fabrication.

Table S1. EXAFS Structural parameters of Te K-edge k^3-weighted EXAFS spectra for S-doped TeO_x samples.

Sample	Scattering path	Energy shift (eV)	Coordination number	Interatomic distance (Å)	Debye-Waller factor (10^{-3} Å^2)	r-factor of fit***
**Te powder (ref.)**	Te-Te	5.54 (±0.74)	2.00*	2.831 (±0.003)**	5.03 (±0.11)	0.0023
**TeO_2 powder (ref.)**	Te-O_1	6.45 (±0.85)	2.00*	1.861 (±0.004)	2.22 (±0.26)	0.0057
	Te-O_2		2.00*	2.108 (±0.005)	4.15 (±0.47)	
**Pristine TeO_x**	Te-O_1	0.93(±1.32)	1.68 (±0.07)	1.863 (±0.008)	5.05 (±0.64)	0.027
	Te-Te		0.99 (±0.04)	2.622 (±0.008)	4.41 (±0.36)	
**S-doped TeO_x**	Te-O_1	2.79 (±1.15)	1.45 (±0.03)	1.878 (±0.004)	4.27 (±0.31)	0.0093
	Te-Te		1.55 (±0.03)	2.781 (±0.004)	7.23 (±0.22)	

* In order to obtain the total amplitude reduction factor, S_0^2, the first shell coordination numbers of reference materials, Te and TeO_2, are fixed to 2. ** Each value in parentheses means the uncertainty obtained from EXAFS fitting process. *** *R*-factor value which is quality of the fit determined with $\{Re\Delta\chi_k^2+Im\Delta\chi_k^2\}/\{Re(\chi_{kdata})^2+Im(\chi_{kdata})^2\}$, where χ(k) is EXAFS-function and $\Delta\chi(k)$ means $\chi(k)_{data} - \chi(k)_{best\text{-}fitted}$.

Table S2. Key parameters of oxide-based ring oscillators and inverters.

Materials	f (kHz)	τ (μs)	RO V_{DD} (V)	stage	inverter V_{DD} (V)	inverter gain	Ref.
SnO/ITZO	/	/	/	/	50	765	13
SnO/IGZO	/	/	/	/	1.5	55	14
TeO_x:Se/IGZO	/	/	/	/	20	1300	27
TeO_x/IGZO	/	/	/	/	2	22	34
SnO/IGZO	8.2	12	4.5	5	3	226	38
SnO/ZnO	2	50	14	5	10	17	45
SnO/ZnO	18.4	5.4	12	5	12	12	46
SnO/IGZO	63	1.2	10	7	10	130	47
SnO/IGZO	2.63	63	40	3	40	24	48
SnO/IGZO	32.87	5.1	20	3	10	110	49
Organic/IZO	77	1.3	10	5	8	36	50
Flexible TeO_x:S/IGZO	328	0.51	12	3	/	/	This work
TeO_x:S/IGZO	339	0.49	12	3	8	1694	This work

REFERENCES AND NOTES

1. K. Nomura, H. Ohta, A. Takagi, T. Kamiya, M. Hirano, H. Hosono, Room-temperature fabrication of transparent flexible thin-film transistors using amorphous oxide semiconductors. *Nature* **432**, 488–492 (2004).

2. J. E. Medvedeva, D. B. Buchholz, R. P. H. Chang, Recent advances in understanding the structure and properties of amorphous oxide semiconductors. *Adv. Electron. Mater.* **3**, 1700082 (2017).

3. S. Lee, A. Nathan, Subthreshold schottky-barrier thin-film transistors with ultralow power and high intrinsic gain. *Science* **354**, 302–304 (2016).

4. H. Çeliker, W. Dehaene, K. Myny, Multi-project wafers for flexible thin-film electronics by independent foundries. *Nature* **629**, 335–340 (2024).

5. A. Nathan, S. Jeon, Oxide electronics: Translating materials science from lab-to-fab. *MRS. Bull.* **46**, 1028–1036 (2021).

6. E. Fortunato, P. Barquinha, R. Martins, Oxide semiconductor thin-film transistors: A review of recent advances. *Adv. Mater.* **24**, 2945–2986 (2012).

7. L. Portilla, K. Loganathan, H. Faber, A. Eid, J. G. D. Hester, M. M. Tentzeris, M. Fattori, E. Cantatore, C. Jiang, A. Nathan, G. Fiori, T. Ibn-Mohammed, T. D. Anthopoulos, V. Pecunia, Wirelessly powered large-area electronics for the Internet of Things. *Nat. Electron.* **6**, 10–17 (2023).

8. X. Yu, T. J. Marks, A. Facchetti, Metal oxides for optoelectronic applications. *Nat. Mater.* **15**, 383–396 (2016).

9. D. G. Georgiadou, J. Semple, A. A. Sagade, H. Forstén, P. Rantakari, Y.-H. Lin, F. Alkhalil, A. Seitkhan, K. Loganathan, H. Faber, T. D. Anthopoulos, 100 GHz zinc oxide Schottky diodes processed from solution on a wafer scale. *Nat. Electron.* **3**, 718–725 (2020).

10. R. Martins, A. Nathan, R. Barros, L. Pereira, P. Barquinha, N. Correia, R. Costa, A. Ahnood, I. Ferreira, E. Fortunato, Complementary metal oxide semiconductor technology with and on paper. *Adv. Mater.* **23**, 4491–4496 (2011).

11. R. F. P. Martins, A. Ahnood, N. Correia, L. M. N. P. Pereira, R. Barros, P. M. C. B. Barquinha, R. Costa, I. M. M. Ferreira, A. Nathan, E. E. M. C. Fortunato, Recyclable, flexible, low-power oxide electronics. *Adv. Funct. Mater.* **23**, 2153–2161 (2013).

12. Z. Wang, P. K. Nayak, J. A. Caraveo-Frescas, H. N. Alshareef, Recent developments in p-type oxide semiconductor materials and devices. *Adv. Mater.* **28**, 3831–3892 (2016).

13. J. Han, R. Hong, H. Liu, W. Niu, X. Yin, L. Tang, S. Zhang, Y. Song, Y. Meng, P. He, H. Huang, L. Tang, X. Liu, X. Zou, L. Liao, High-mobility SnO enabled by doping-induced interstitial oxygen for all-oxide complementary logics. *Adv. Funct. Mater.* **35**, 2500132 (2025).

14. J. Lu, M. Shen, X. Feng, T. Tan, H. Guo, L. Lin, F. Zhou, Y. Li, P-type oxide thin-film transistor with unprecedented hole field-effect mobility for an all-oxide CMOS CFET-like inverter suitable for monolithic 3D integration. *Nano Lett.* **24**, 15260–15267 (2024).

15. R. Barros, K. J. Saji, J. C. Waerenborgh, P. Barquinha, L. Pereira, E. Carlos, R. Martins, E. Fortunato, Role of structure and composition on the performances of p-type tin oxide thin-film transistors processed at low-temperatures. *Nanomaterials* **9**,320 (2019).

16. E. Fortunato, R. Barros, P. Barquinha, V. Figueiredo, S.-H. K. Park, C.-S. Hwang, R. Martins, Transparent p-type SnO_x thin film transistors produced by reactive RF magnetron sputtering followed by low temperature annealing. *Appl. Phys. Lett.* **97**, 052105 (2010).

17. J. Yang, Y. Yuan, Y. Li, L. Du, Y. Wang, Z. Hu, Q. Wang, L. Zhou, Q. Xin, A. Song, All-oxide-semiconductor-based thin-film complementary static random access memory. *IEEE Electron. Device. Lett.* **39**, 1876–1879 (2018).

18. R. Hong, P. He, S. Zhang, X. Hong, Q. Tian, C. Liu, T. Bu, W. Su, G. Li, D. Flandre, X. Liu, Y. Lv, L. Liao, X. Zou, Compositional engineering of Cu-doped SnO film for complementary metal oxide semiconductor technology. *Nano Lett.* **24**, 1176–1183 (2024).

19. Y. Wang, G. Qiu, R. Wang, S. Huang, Q. Wang, Y. Liu, Y. Du, W. A. Goddard, M. J. Kim, X. Xu, P. D. Ye, W. Wu, Field-effect transistors made from solution-grown two-dimensional tellurene. *Nat. Electron.* **1**, 228–236 (2018).

20. C. Zhao, C. Tan, D.-H. Lien, X. Song, M. Amani, M. Hettick, H. Y. Y. Nyein, Z. Yuan, L. Li, M. C. Scott, A. Javey, Evaporated tellurium thin films for p-type field-effect transistors and circuits. *Nat. Nanotechnol.* **15**, 53–58 (2020).

21. R. An, Y. Li, J. Tang, B. Gao, Y. Du, J. Yao, Y. Li, W. Sun, H. Zhao, J. Li, Q. Qin, Q. Zhang, S. Qiu, Q. Li, Z. Li, H. Qian, H. Wu, A hybrid computing-in-memory architecture by monolithic 3D integration of BEOL CNT/IGZO-based CFET logic and analog RRAM, in *2022 International Electron Devices Meeting* (IEEE, 2022), pp. 18.11.11-18.11.14.

22. Q. He, Y. Liu, C. Tan, W. Zhai, G.-h. Nam, H. J. A. N. Zhang, Quest for p-type two-dimensional semiconductors. *ACS Nano* **13**, 12294–12300 (2019).

23. X. Xu, Y. Pan, S. Liu, B. Han, P. Gu, S. Li, W. Xu, Y. Peng, Z. Han, J. J. S. Chen, Seeded 2D epitaxy of large-area single-crystal films of the van der Waals semiconductor 2H $MoTe_2$. *Science* **372**, 195–200 (2021).

24. S. Jana, E. Carlos, S. Panigrahi, R. Martins, E. Fortunato, Toward stable solution-processed high-mobility p-type thin film transistors based on halide perovskites. *ACS Nano* **14**, 14790–14797 (2020).

25. A. Liu, H. Zhu, S. Bai, Y. Reo, M. Caironi, A. Peterzza, L. Dou, Y.-Y. Noh, High-performance metal halide perovskite transistors, *Nat. Electron.* **6**, 559–571 (2023).

26. H. Zhu, W. Yang, Y. Reo, G. Zheng, S. Bai, A. Liu, Y.-Y. Noh, Tin perovskite transistors and complementary circuits based on A-site cation engineering, *Nat. Electron.* **6**, 650–657 (2023)

27. A. Liu, Y.-S. Kim, M. G. Kim, Y. Reo, T. Zou, T. Choi, S. Bai, H. Zhu, Y.-Y. Noh, Selenium-alloyed tellurium oxide for amorphous p-channel transistors. *Nature* **629**, 798–802 (2024).

28. J. Robertson, X. Zhang, Q. Gui, Y. Guo, Amorphous TeO_2 as p-type oxide semiconductor for device applications. *Appl. Phys. Lett.* **124**, 212101 (2024).

29. P. Tan, C. Niu, Z. Lin, J.-Y. Lin, L. Long, Y. Zhang, G. Wilk, H. Wang, P. D. Ye, Wafer-scale atomic layer-deposited TeO_x/Te heterostructure p-type thin-film transistors. *Nano Lett.* **24**, 12433–12441 (2024).

30. Y. Zhang, J. Wang, P. Xie, Y. Meng, H. Shao, C. Jin, B. Gao, Y. Shen, Q. Quan, Y. Li, W. Wang, D. Li, Z. Wu, B. Li, S.P. Yip, J. Sun, J. C. Ho, Molecular reconfiguration of disordered tellurium oxide transistors with biomimetic spectral selectivity. *Adv. Mater.* **36** e2412210 (2024).

31. T. Kim, C. H. Choi, S. E. Kim, J.-K. Kim, J. Jang, S. Choi, J. Noh, K.-S. Park, J. Kim, S. Y. Yoon, J. K. Jeong, High-performance hexagonal tellurium thin-film transistor using tellurium oxide as a crystallization retarder. *IEEE Electron. Device Lett.* **44**, 269–272 (2022).

32. Y. Xu, Y. Sun, Z. Zheng, Y. Wang, Y. Kang, K. Han, X. Chen, G. Zheng, X. Gong, BEOL-compatible Te-TeO_x p-FETs with channel length down to 50 nm at cryogenic temperatures. *Appl. Phys. Lett.* **126**, 033502 (2025).

33. Y. Su, T. Liu, J. Tang, Y. Li, R. An, Y. Du, Z. Tang, Y. Zhang, Y. Fan, Y. He, M. Shi, H. Yang, T. Huang, J. Zhang, Z. Zhu, G. Wang, C. Zhao, C. Wang, L. Pan, P. Yao, D. Wu, B. Gao, H. Qian, H. Wu, Complementary oxide semiconductor-based 2T0C DRAM macro with CFET peripherals using TeOx-PFET/IGZO-NFET for 3D Memory Integration, in *2024 International Electron Devices Meeting (IEDM)* (IEEE, 2024), pp. 1–4.

34. T. Liu, J. Tang, Y. Du, H. Yang, Y. Zhang, Z. Liu, Z. Jiang, R. An, Y. Xi, Y. Li, D. Wu, B. Gao, H. Qian, H. Wu, IGZO/TeO_x complementary oxide semiconductor-based CFET for BEOL-compatible memory-immersed logic, in *2024 International Electron Devices Meeting (IEDM)* (IEEE, 2024), pp. 1–4.

35. C. K. Kang, S. U. Yuldashev, J. H. Leem, Y. S. Ryu, J. K. Hyun, H. S. Jung, H. J. Kim, T. W. Kang, H. I. Lee, Y. D. Woo, T. W. Kim, Surface passivation by sulfur treatment of undoped p-CdTe (100). *J. Appl. Phys.* **88**, 2013–2015 (2000).

36. P. Lee, M. N. Le, G. Kim, S. M. Kwon, J.-W. Jo, J. Kim, Y.-H. Kim, S. K. Park, K. Ahn, M.-G. Kim Efficient Oxygen‐Vacancy Suppression and Electrical Stabilization of Solution‐Processed In2O3:Q (Q=S, Se) Thin‐Film Transistor with Chalcogen Alloying. *Adv. Electron. Mater.* **8**, 2101250 (2022).

37. K. Myny, The development of flexible integrated circuits based on thin-film transistors. *Nat. Electron.* **1**, 30–39 (2018).

38. Y.-S. Li, J.-C. He, S.-M. Hsu, C.-C. Lee, D.-Y. Su, F.-Y. Tsai, I.-C. Cheng, Flexible complementary oxide–semiconductor-based circuits employing n-channel ZnO and p-channel SnO thin-film transistors. *IEEE Electron Device Lett.* **37**, 46–49 (2016).

39. Y. Chen, D. Geng, T. Lin, M. Mativenga, J. Jang, Full-swing clock generating circuits on plastic using a-IGZO dual-gate TFTs with pseudo-CMOS and bootstrapping. *IEEE Electron Device Lett.* **37**, 882–885 (2016).

40. G. Kresse, J. Furthmüller, Efficient iterative schemes for ab initio total-energy calculations using a plane-wave basis set. *Phys. Rev. B* **54**, 11169–11186 (1996).

41. P. E. Blöchl, Projector augmented-wave method. *Phys. Rev. B* **50**, 17953–17979 (1994).

42. G. Kresse, D. Joubert, From ultrasoft pseudopotentials to the projector augmented-wave method. *Phys. Rev. B* **59**, 1758–1775 (1999).

43. J. P. Perdew, K. Burke, M. Ernzerhof, Generalized gradient approximation made simple. *Phys. Rev. Lett.* **77**, 3865–3868 (1996).

44. J. Heyd, G. E. Scuseria, M. Ernzerhof, Ernzerhof, Hybrid functionals based on a screened Coulomb potential. *J. Chem. Phys.* **118**, 8207–8215 (2003).

45. I.C. Chiu, Y.S. Li, M.S. Tu, I.C. Cheng, Complementary oxide-semiconductor-based circuits with n-channel ZnO and p-channel SnO thin-film transistors, *IEEE Electron Device Lett.* **35**, 1263–1265 (2014).

46. Y. Yuan, J. Yang, Z. Hu, Y. Li, L. Du, Y. Wang, L. Zhou, Q. Wang, A. Song, Q. Xin, Oxide-based complementary inverters with high gain and nanowatt power consumption, *IEEE Electron Device Lett.* **39**, 1676–1679 (2018).

47. Y. Li, J. Zhang, J. Yang, Z. Hu, Z. Lin, A. Song, Q. Xin, Complementary integrated circuits based on n-type and p-type oxide semiconductors for applications beyond flat-panel displays, *IEEE Trans. Electron Devices* **66**, 950–956 (2019).

48. J. Zhang, J. Yang, Y. Li, J. Wilson, X. Ma, Q. Xin, A. Song, High performance complementary circuits based on p-SnO and n-IGZO thin-film transistors, *Materials* **10** 319 (2017).

49. Y. Li, J. Yang, Y. Wang, P. Ma, Y. Yuan, J. Zhang, Z. Lin, L. Zhou, Q. Xin, A. Song, Complementary integrated circuits based on p-type SnO and n-type IGZO thin-film transistors, *IEEE Electron Device Lett.* **39**, 208–211 (2018).

50. X. Wei, S. Kumagai, T. Makita, K. Tsuzuku, A. Yamamura, M. Sasaki, S. Watanabe, J. Takeya, High-speed hybrid complementary ring oscillators based on solution-processed organic and amorphous metal oxide semiconductors, *Commun. Mater.* **4**, 4 (2023).
